# Supplementary material for: Paclitaxel-induced tubulin dysfunction stalls parasite development: synergistic potential with artemisinin against resistant strains
Source: Microbiol Spectr. 2026 Apr 30;14(6):e01957-25. doi: 10.1128/spectrum.01957-25 (PMC13227983; doi:10.1128/spectrum.01957-25)
Supplement: Supplemental material — Fig. S1; Table S1. [file spectrum.01957-25-s0001.docx]

**Paclitaxel-Induced Tubulin Dysfunction Stalls Parasite Development: Synergistic Potential with Artemisinin against Resistant Strains**

Yongxin Tang^1,2 #^, Xinyu Zhang^1,2, #^, Xiaohui He^2, #^, Tao Jiang^3,^ *, Jun Cao^1,2,^ *, Xinyu Yu^2,^ *

^1^ Center for Global Health, School of Public Health, Nanjing Medical University, Nanjing, China.

^2^ National Health Commission Key Laboratory of Parasitic Disease Control and Prevention, Jiangsu Provincial Key Laboratory on Parasite and Vector Control Technology, Jiangsu Institute of Parasitic Diseases, Wuxi, China.

^3^ State Key Laboratory of Biomacromolecules, Institute of Biophysics, Chinese Academy of Science, Beijing, China

# These authors contribute equally to this work.

* Corresponding authors.

Email addresses: [Kimiyxy@foxmail.com](mailto:Kimiyxy@foxmail.com) (X. Yu), [caojuncn@hotmail.com](mailto:caojuncn@hotmail.com) (J. Cao), tjiang@ibp.ac.cn (T. Jiang)

**Artemnisinin-resustance induction for *P. berghei***

*P. berghei* ART-resistant strains were generated using a repeated drug selection protocol as previously described(1, 2). Briefly, female BALB/c mice (6-8 weeks old) were intraperitoneally infected with the parental *P. berghei* strain. Subsequently, the infected mice were randomly allocated to four experimental groups (n=3): three ART treatment groups with graded dosages (1.25, 2.5, and 5 mg/kg body weight) and one vehicle control group. All treatments were administered consecutively for 4 days, consistent with the standard 4-day suppressive assay for antimalarial drug evaluation. Parasitemia was quantitatively determined daily via microscopic examination of Giemsa-stained thin blood smears. The 50% effective dose (ED₅₀) and 90% effective dose (ED₉₀) against each parasite line were calculated using a linear regression model based on the parasite inhibition rate. The resistance index (I₉₀) was defined as the ratio of ED₉₀ values between the drug-selected resistant strain and the parental sensitive strain. According to previously established criteria, parasite strains were categorized into four resistance grades based on I₉₀ values: sensitive (I₉₀ = 1.0), slight resistance (I₉₀ = 1.01-10.0), moderate resistance (I₉₀ = 10.01-100.0), and high resistance (I₉₀ > 100.0)(3).

After determining the ED₅₀ and ED₉₀ of the parental parasite strain, an additional 3 female BALB/c mice were intraperitoneally infected with the same parental strain. Parasitemia was monitored quantitatively via daily microscopic examination of Giemsa-stained thin blood smears until it reached 3%-5%. The mice were then treated with ART at the ED₉₉ dosage, and the resistance level was assessed every 10 passages (10 cycles) using the 4-day suppressive assay to determine the updated ED₅₀ and ED₉₀ values.

This drug selection and resistance monitoring process was repeated continuously for 30 consecutive passages to ensure the establishment of a genetically stable ART-resistant *P. berghei* strain. The resistance phenotype of the parasite strains was quantitatively characterized and the corresponding data are summarized in Supplementary Table S1. Following the successful generation of this resistant strain, the *in vivo* antimalarial activity of PTX against the strain was evaluated as described above.

**Table. S1** Response of ART resistant P. berghei ANKA line to dihydroartemisinin (DHA). Results are presented as the 50% and 90% resistance indexes (I₅₀, I₉₀), defined as the ratio of the 50% effective dose (ED₅₀) or 90% effective dose (ED₉₀) of the resistant line to those of the isogenic drug-sensitive parental strain.

| **Passage No.** | **ED_50_ (mg/kg)** | **I_50_** | **ED_90_ (mg/kg)** | **I_90_** |
| --- | --- | --- | --- | --- |
| Parent | 0.97 | N.A. | 3.48 | N.A. |
| 10 | 1.59 | 1.639 | 4.33 | 1.244 |
| 20 | 2.77 | 2.856 | 10.39 | 2.986 |
| 30 | 3.32 | 3.423 | 12.21 | 3.509 |

**Effect of PTX esposure on the parasite normal growth during IDC**

To rule out the potential growth arrest effect induced by PTX treatment, we conducted additiopnal experiments using gametocytes exposed to 500 nM PTX for two consecutive days (on Day 1 and Day 7 post-induction). Gametocytes at distinct developmental stages were quantified to clarify whether PTX treatment exerts a lethal effect on the parasites or merely arrests their growth. Our results showed that a mild growth arrest was observed immediately after PTX exposure. However, to assess whether this exposure would trigger long-term growth arrest during gametocyte maturation, we compared the percentage of gametocytes at each developmental stage at Days 3, 6, 9, 12 and 15 between the PTX-treated group and the control group. Following one-way ANOVA with Bonferroni correction, no statistically significant differences were observed between the two groups. This finding demonstrates that PTX treatment leads to parasite death rather than simply arresting their growth.

Fig. S1 Gametocyte developmental dynamics over time in the control group (A), and in groups treated with PTX on Day 1 (B) and Day 7 (C). Each bar represents the percentage of a specific gametocyte stage relative to the total number of gametocytes counted. Percentage of gametocytes at each developmental stage on Days 3, 6, 9, 12 and 15. No statistical significance was observed between the groups.


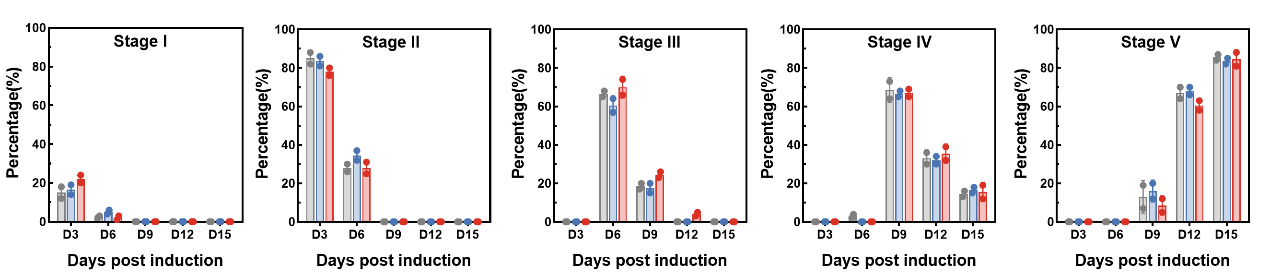

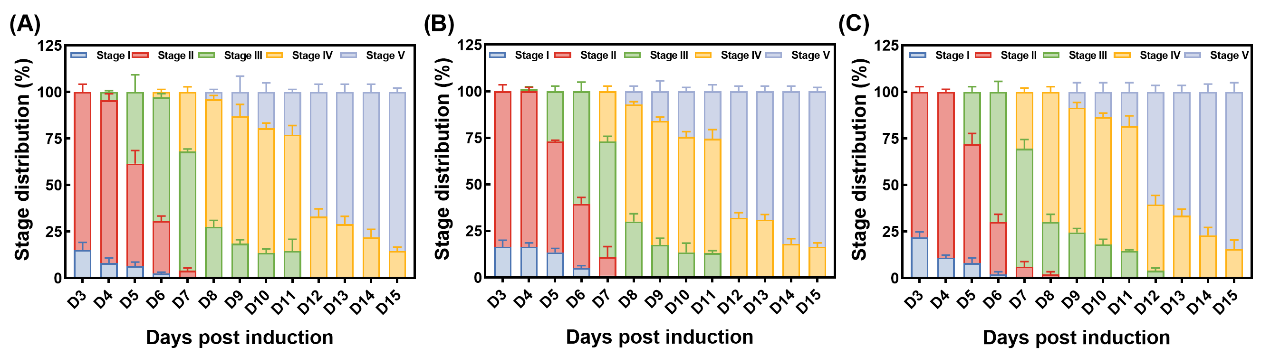


1. Kiboi D, Irungu B, Langat B, Wittlin S, Brun R, Chollet J, Abiodun O, Nganga J, Nyambati V, Rukunga G. 2009. Plasmodium berghei ANKA: Selection of resistance to piperaquine and lumefantrine in a mouse model. Experimental parasitology 122:196-202.

2. Xiao S-H, Yao J-M, Utzinger J, Cai Y, Chollet J, Tanner M. 2004. Selection and reversal of Plasmodium berghei resistance in the mouse model following repeated high doses of artemether. Parasitology Research 92:215-219.

3. Merkli B, Richle R, Peters W. 1980. The inhibitory effect of a drug combination on the development of mefloquine resistance in Plasmodium berghei. Annals of Tropical Medicine & Parasitology 74:1-9.
